# Data on bulk rock compositions, geochemical and textural contrasts between central and marginal parts of dykes, and MELTS modeling of lamprophyre dykes in the Kola Alkaline Carbonatite Province (N Europe)

**DOI:** 10.1016/j.dib.2021.107307

**Published:** 2021-08-19

**Authors:** A.A. Nosova, M.G. Kopylova, L.V. Sazonova, A.A. Vozniak, N.M. Lebedeva, G.D. Volkova, E.V. Peresetskaya

**Affiliations:** a Vernadsky Institute of Geochemistry and Analytical Chemistry of Russian Academy of Sciences, Kosygina 19, Moscow 119991, Russia; b Institute of Geology of Ore Deposits, Petrography, Mineralogy, and Geochemistry, Russian Academy of Sciences (IGEM RAS), Staromonetny 35, Moscow 119017, Russia; c The University of British Columbia, 2207 Main Mall, Vancouver, BC V6T1Z4, Canada; d Geological Department, Lomonosov Moscow State University, GSP-1, Leninskie Gory, Moscow, Russia

**Keywords:** Lamprophyric dykes, Kola Alkaline Carbonatite Province, Crystal fractionation, Core-margin dyke heterogeneity, MELTS modeling, Liquid immiscibility

## Abstract

This publication is a series of datasets that accompany a manuscript on petrology of lamprophyre dykes in the Kola Alkaline Carbonatite Province (N Europe) [1]. The datasets served as the basis for interpretation of melt crystallization for lamprophyric and carbonatitic dykes in the crust, to supplement many papers devoted to mantle sources and melting parameters of these rocks based on radiogenic isotopes and trace elements. The first dataset contains bulk major and trace element compositions of the dykes in three areas, Kandalaksha, Kandaguba and Turiy Mys, along with supplementary information on sampling locations and dyke characteristics. The second dataset represents photos, major and trace element plots illustrating extreme mineralogical and textural heterogeneity and layering observed in some dykes. The photographs include field photographs, optical and electron microscopy shots for thin sections in central and marginal parts of the dykes. They should be viewed together with Harker and REE diagrams illustrating the changes from dyke margins to dyke cores. The third and fourth datasets are output tables from Ryolite-MELTS software used to model crystal fractionation of the dykes from the parental melts and various P, T, fO_2_, XCO_2_, XH_2_O parameters. One table shows compositions of evolved melts, while the other lists crystallizing phases.

**Specifications Table**

**Table:** 

Subject	Geochemistry and Petrology
Specific subject area	Petrology of lamprophyres and carbonatites
Type of data	Tables Images Figures Graphs
How data were acquired	Field photographs in geographical areas specified in Table 1; Optical photographs on thin sections on a polarizing microscope; BSE photos on a JEOL JSM-6480LV scanning electron microscope (SEM); X-ray fluorescence (XRF) spectroscopy and Laser Ablation Inductively Coupled Plasma Mass Spectrometry (ICP-MS) on rock powders; Modeling on the Rhyolite-MELTS software package (version 1.2.0, [2])
Data format	Raw analyzed
Parameters for data collection	SEM photography was performed at 20 kV and beam current 20 ηA. X-ray fluorescence analyses were determined using a Philips Analytical B.V. PW-2400 spectrometer. Laser Ablation Inductively Coupled Plasma Mass Spectrometry (ICP-MS) was carried out at the Institute of Microelectronics Technology and High Purity Materials (RAS, Chernogolovka) using analytical protocols of [3]. Modeling on the Rhyolite-MELTS was done for melanephelinite and magnesian aillikite, alnöite, monchiquite, at 1.3–0.5 GPa and 200–10 MPa. The modeling was carried out at Δlog fO_2_ (QFM)=0–2, overall fluid content 7 wt%, CO_2_/H_2_O ratios from 2/5 to 5/2, at equilibrium and fractional crystallization conditions.
Description of data collection	For XRF analysis the fused glass disks were prepared by melting 0.3 g of the powdered sample with 3 g of lithium tetraborate in an induction furnace. For ICP-MS analysis, rock powders were treated with inorganic acids in a sealed autoclave with the sample decomposition monitored by addition of ^161^Dy.
Data source location	Kola peninsula Country: Russian Federation GPS coordinates for collected samples/data are given in Table 1 and in Electronic Supplement 1
Data accessibility	The data are hosted with the article
Related research article	Nosova, A. A., Kopylova, M. G., Sazonova, L. V., Vozniak, A. A., Kargin, A. V., Lebedeva, N. M., Volkova, G. D., Peresetskaya, E.V. (2021) Petrology of lamprophyre dykes in the Kola Alkaline Carbonatite Province (N Europe). Lithos, Volumes 398–399, October 2021, https://doi.org/10.1016/j.lithos.2021.106277

**Value of the Data**

• This collection of tables, plots and images provides new data on lamprophyric and carbonatitic Devonian dykes in three areas of the Kola Alkaline Carbonatite Province, Kandalaksha, Kandaguba and Turiy Mys.
• This Data-in-Brief is the supplementary information for a companion paper [1] that highlights the critical role of the parent magma chamber in crystal fractionation and magma diversification.
• The value of the major and trace element chemistry dataset is in thorough petrographic examinations of all geochemical samples. Previous papers that reported on Kola dykes either (1) did not place the geochemical work on the solid ground of petrography; or (2) provided the minimal petrography, or (3) reported petrography with arbitrary rock names not approved by IUGS [4] or (4) did not provide SEM photographs or mineral modes that allows a re-classification of the rocks after a new classification of ultramafic lamprophyres [5] became available. Our unique emphasis on the petrography is especially valuable as the bulk composition alone cannot provide a correct name for lamprophyres; the latter are distinguished by the modal mineralogy and texture [4].
• The dataset on textural, mineralogical and geochemical changes from marginal to central parts of thin dykes provides rarely reported information. The petrographic contrast and significant shifts in the major and trace element compositions exceeds those commonly reported for thin, 10–30 cm dykes. The observed mineralogical contrasts in dykes on the scale of tens of centimeters, in our opinion, are phenomena related to the complex behavior of the fluid, and should be as typical of lamprophyres as ocelli.
• Mantle source rocks and melting parameters for rocks in ultramafic–alkaline-carbonatite dyke series are well explored, but their crustal crystallization, including the order of crystallization and conditions of crystallization in intermediate magma chambers and on the surface are poorly known. The value of data on MELTS fractionation modeling is in constraints on the crystallization behavior during the ascent of the melts in the crust.
• Petrologists, mineralogists and geochemists can benefit from the data, as the studied dykes could be seen as an example of an association of alkaline ultramafic-to-mafic rocks with carbonatites that occur repeatedly over time and space. The data on the center-to-margin contrasts within the dykes can be used for further insights about the rock origin in a spatially and temporally linked rock series. The data on MELTS modeling can be used for theoretical sequences of crystallization and liquid lines of descent at a variety of parameters realistically expected for lamprophyre crystallization.

## 1 Data Description

Table 1 entitled “Bulk major and trace element composition of studied KACP dykes” is a catalogue of all collected samples with GPS coordinates, lithologies and bulk major and trace element analyses. The Table is provided as a raw data Electronic Supplementary File 1 and can also be seen as a PDF table in this publication.

Figs. 1–8 illustrate extreme mineralogical and textural heterogeneity and layering observed in some dykes.

**Fig. 1:**
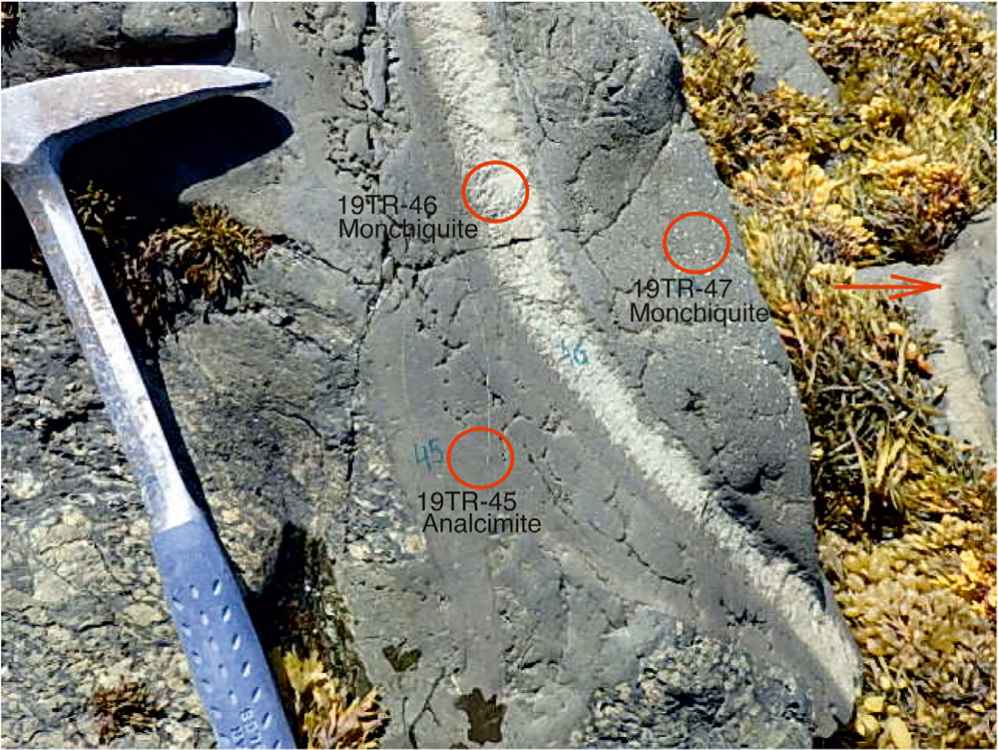
A field photo of a layered Turiy Mys dyke with samples 19TR-45, 46, 47. Aphyric chilled margin 19TR-45 transitions to white, 3 cm-thin rhythmic layering parallel to the dyke strike (19TR-46) and then to porphyritic sample 19TR-47. The dyke continues further to the right, where another white layer of the rhythmic layering is marked with an arrow.

**Fig. 2:**
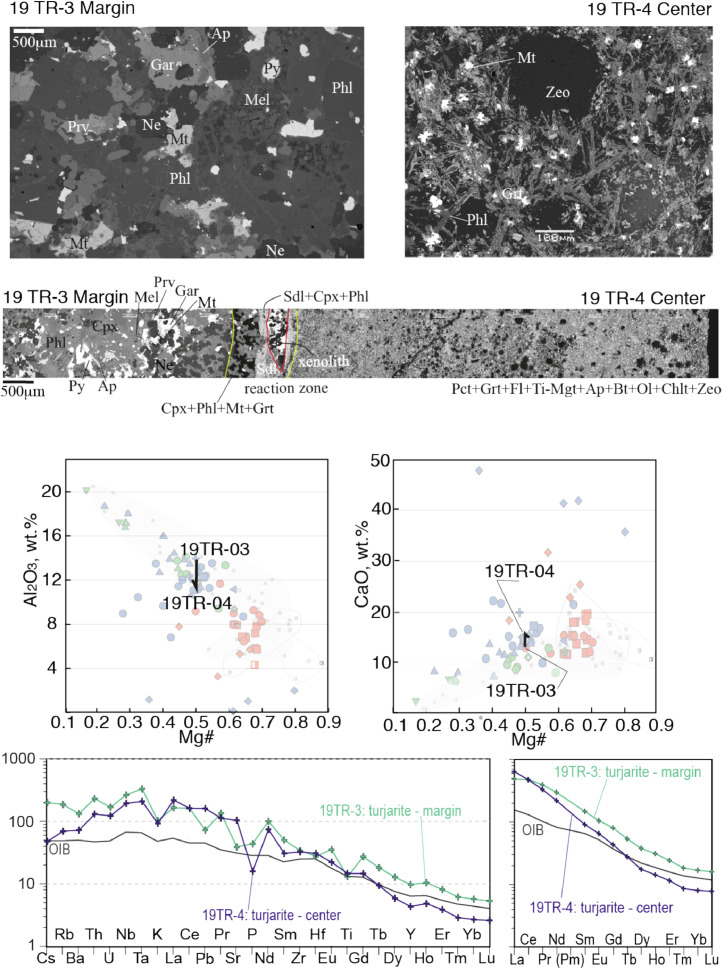
Contrasting petrography, major and trace element compositions of central (19TR-4) and marginal parts (19TR-3) of a turjaite dyke. The upper three microphotographs are backscattered electron images made on SEM. Mineral abbreviations are Mt- magnetite, Zeo-zeolite, Grt - garnet, Ti-Mt - Ti-magnetite, Phl - phlogopite, Ap -apatite, Mel- melilite, Py - pyrite, Ne- nepheline, Prv- perovskite, Sdl sodalite, Fl - fluorite, Cpx-clinopyroxene, Pct - pectolite, Bt - biotite, Ol- olivine, Chlt chlorite. Below the microphotographs two plots show SiO2 Mg# and CaO - Mg# plots for bulk rock compositions of central and marginal parts of the dykes, with arrows that lead from the margin to the core. The background for the major element diagrams are plots from Fig. 8 of [1], where red symbols denote Kandalaksha, green symbols for Kandaguba, blue symbols for Turiy Mys, small symbols and open fields mark bulk compositional field for the Khibina dykes and massif [6], [7]. The lower two plots are trace element bulk rock compositions of central and marginal parts of the dyke normalized to the primitive mantle [8] and compared with the OIB patterns [8].

**Fig. 3:**
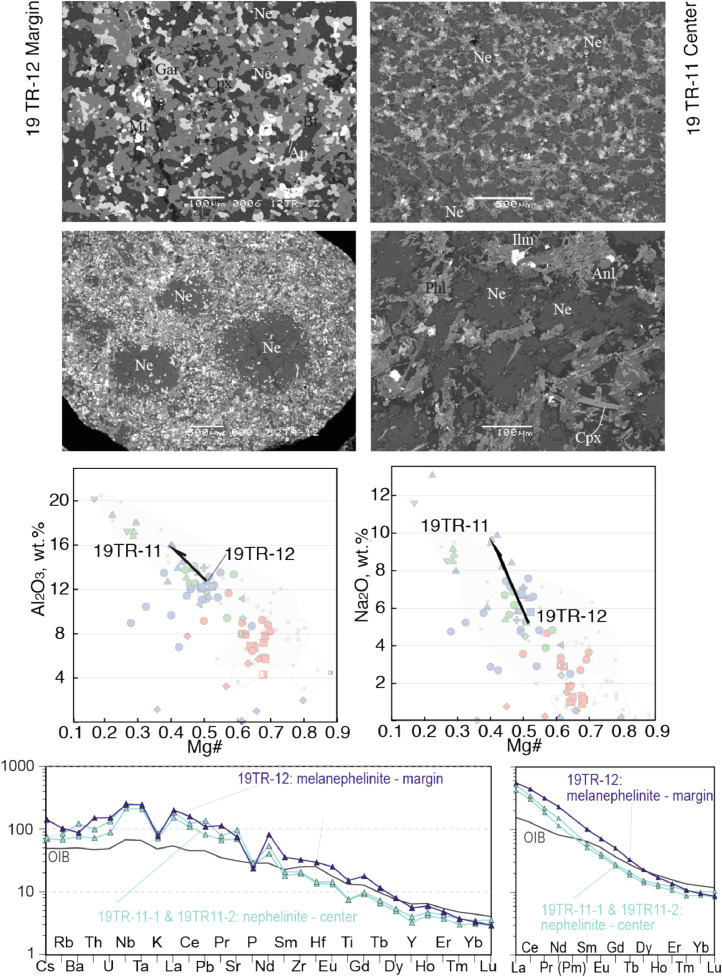
Contrasting petrography, major and trace element compositions of central (19TR-11) and marginal parts (19TR-12) of a nephelinite-melanephelinite dyke. The upper four microphotographs are backscattered electron images made on SEM. Mineral abbreviations are Cb - carbonate, Anl analcime, Mt- magnetite, Grt - garnet, Phl - phlogopite, Ap -apatite, Ne- nepheline, Cpx-clinopyroxene, Bt biotite. Below the microphotographs two plots show Al2O3 Mg# and Na2O - Mg# plots for bulk rock compositions of central and marginal parts of the dykes, with arrows that lead from the margin to the core. The background for the major element diagrams are plots from Fig. 8 of [1], where red symbols denote Kandalaksha, green symbols for Kandaguba, blue symbols for Turiy Mys, small symbols and open fields mark bulk compositional field for the Khibina dykes and massif [6], [7]. The lower two plots are trace element bulk rock compositions of central and marginal parts of the dyke normalized to the primitive mantle [8] and compared with the OIB patterns [8].

**Fig. 4:**
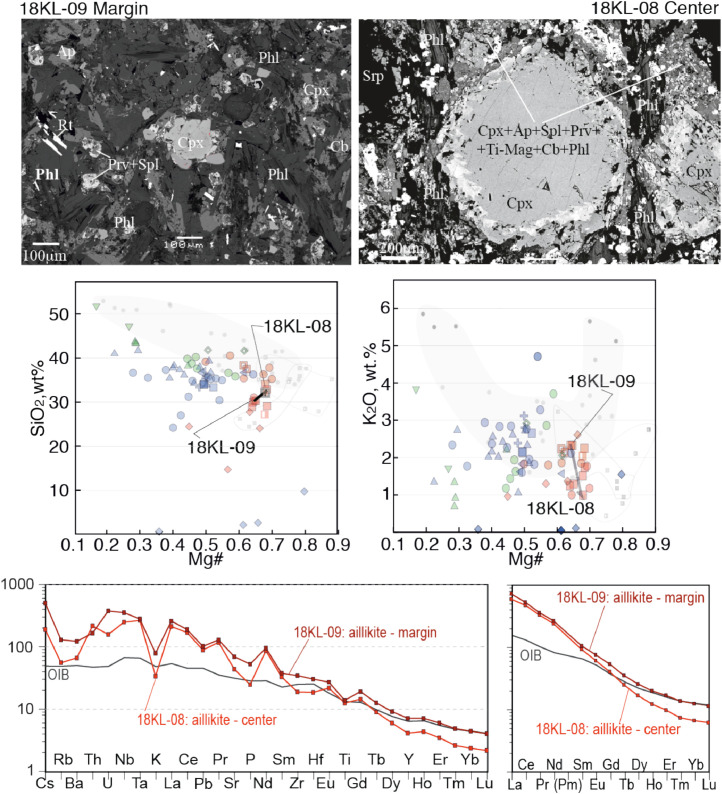
Contrasting petrography, major and trace element compositions of central (19KL-08) and marginal parts (19KL-09) of an aillikite dyke. The upper two microphotographs are backscattered electron images made on SEM. Mineral abbreviations are Cb - carbonate, Anl – analcime, Mt- magnetite, Spl- spinel, Grt - garnet, Phl - phlogopite, Ap - apatite, Ne- nepheline, Cpx-clinopyroxene, Bt – biotite. Ti-Mt - Ti-magnetite, Prv- perovskite, Rt- rutile. Below the microphotographs two plots show SiO_2_ – Mg# and K_2_O - Mg# plots for bulk rock compositions of central and marginal parts of the dykes, with arrows that lead from the margin to the core. The background for the major element diagrams are plots from Fig. 8 of [1], where red symbols denote Kandalaksha, green symbols for Kandaguba, blue symbols for Turiy Mys, small symbols and grey fields mark bulk compositional field for the Khibina dykes and massif [6], [7]. The lower two plots are trace element bulk rock compositions of central and marginal parts of the dyke normalized to the primitive mantle [8] and compared with the OIB patterns [8].

**Fig. 5:**
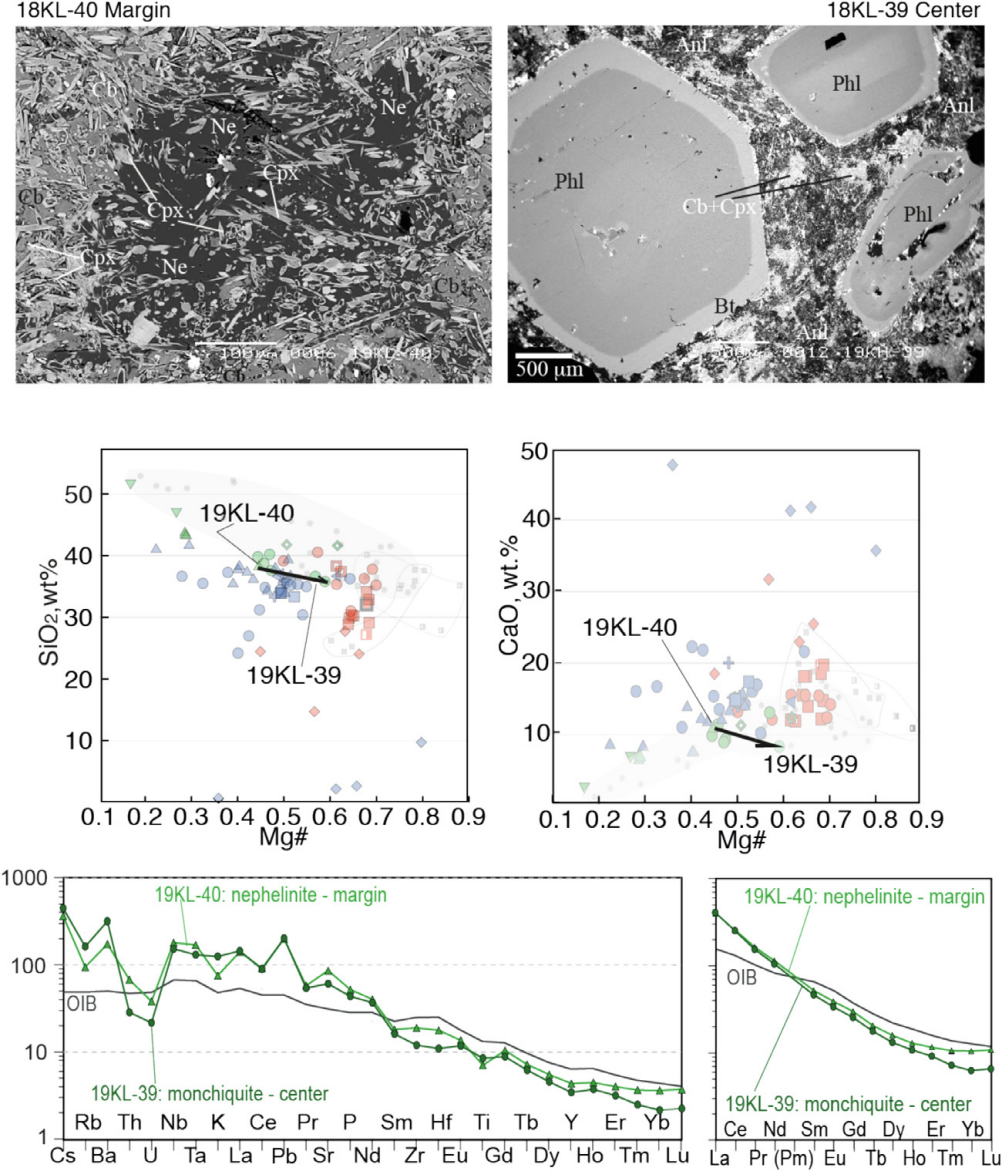
Contrasting petrography, major and trace element compositions of central (19KL-39) and marginal parts (19KL-40) of a nephelinite-monchiquite dyke. The upper two microphotographs are backscattered electron images made on SEM. Mineral abbreviations are Cb - carbonate, Anl analcime, Phl - phlogopite, Ne- nepheline, Cpx-clinopyroxene, Bt biotite. Below the microphotographs two plots show SiO2 Mg# and CaO - Mg# plots for bulk rock compositions of central and marginal parts of the dykes, with arrows that lead from the margin to the core. The background for the major element diagrams are plots from Fig. 8 of [1], where red symbols denote Kandalaksha, green symbols for Kandaguba, blue symbols for Turiy Mys, small symbols, grey and open fields mark bulk compositional field for the Khibina dykes and massif [6], [7]. The lower two plots are trace element bulk rock compositions of central and marginal parts of the dyke normalized to the primitive mantle [8] and compared with the OIB patterns [8].

**Fig. 6:**
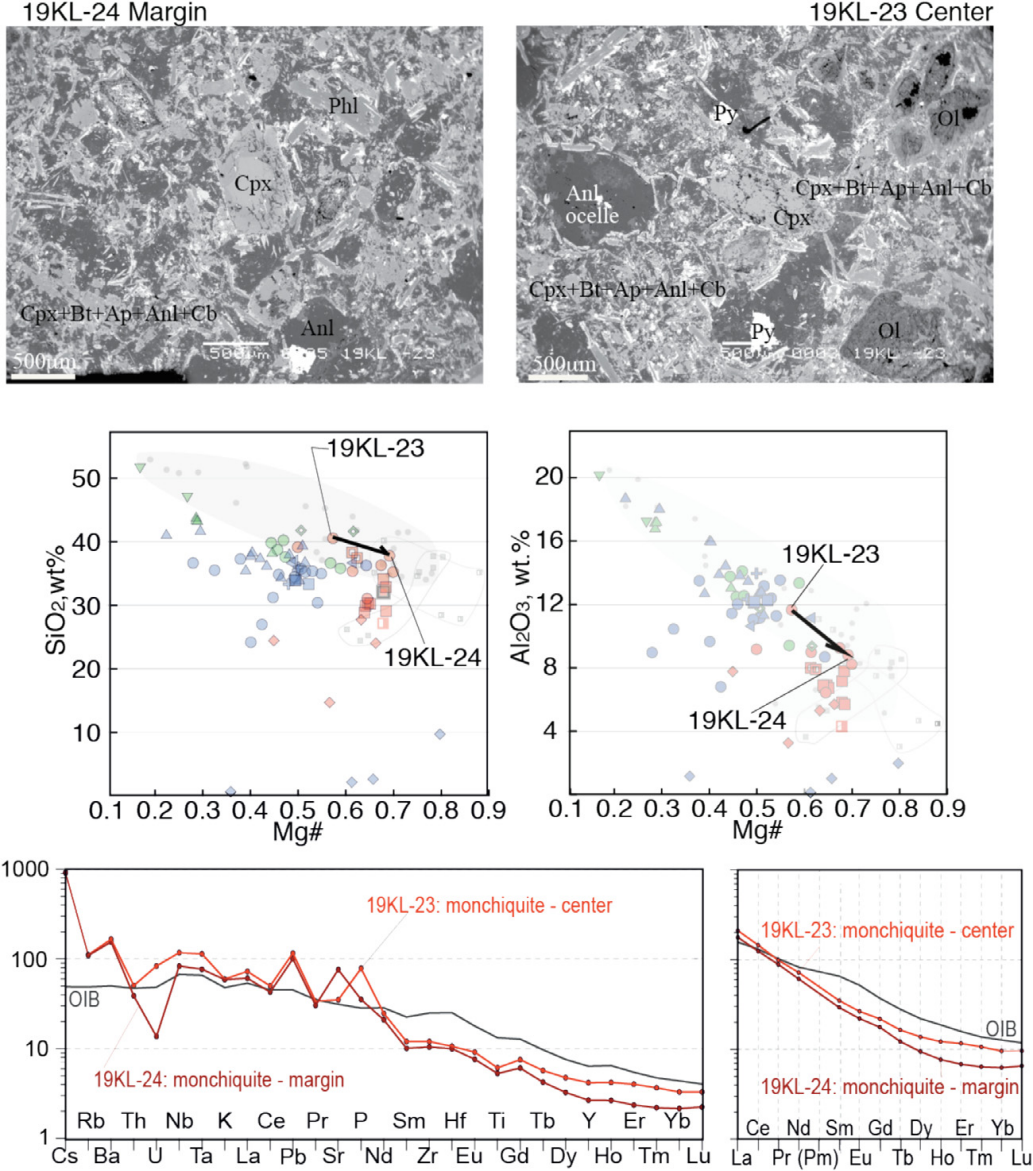
Contrasting petrography, major and trace element compositions of central (19KL-23) and marginal parts (19KL-24) of a monchiquite dyke. The upper two microphotographs are backscattered electron images made on SEM. Mineral abbreviations are Ol – olivine, Py - pyrite, Cb - carbonate, Ap-apatite, Anl – analcime, Phl - phlogopite, Cpx-clinopyroxene, Bt – biotite. Below the microphotographs two plots show SiO_2_ – Mg# and Al_2_O_3_ - Mg# plots for bulk rock compositions of central and marginal parts of the dykes, with arrows that lead from the margin to the core. The background for the major element diagrams are plots from Fig. 8 of [1], where red symbols denote Kandalaksha, green symbols for Kandaguba, blue symbols for Turiy Mys, small symbols and grey fields mark bulk compositional field for the Khibina dykes and massif [6], [7]. The lower two plots are trace element bulk rock compositions of central and marginal parts of the dyke normalized to the primitive mantle [8] and compared with the OIB patterns [8].

**Fig. 7:**
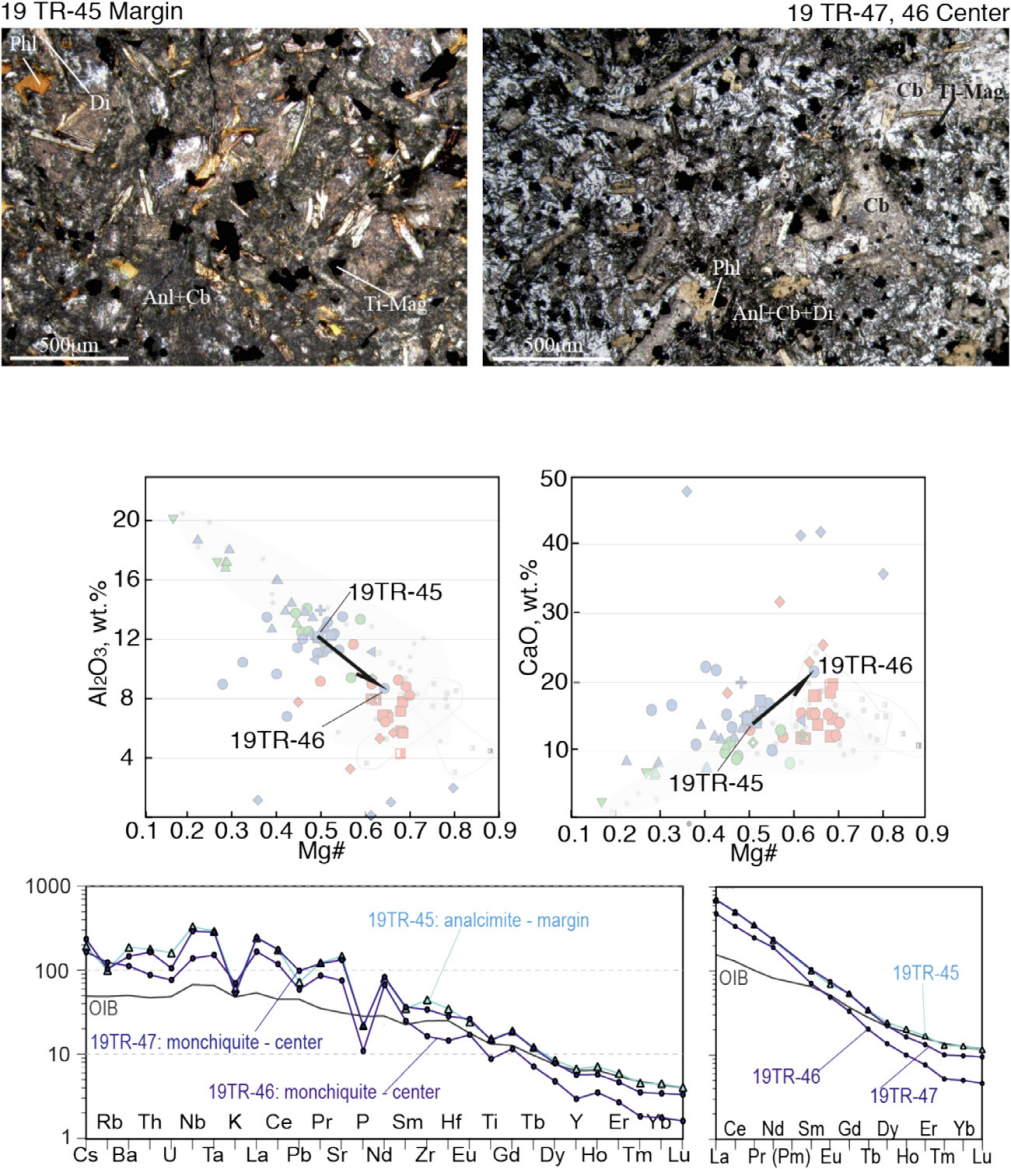
Contrasting petrography, major and trace element compositions of central (19TR-46, 47) and marginal parts (19TR-45) of a monchiquite-analcimite dyke. The upper two microphotographs are optical plain polarized light microscopic images. Mineral abbreviations are Ti-Mt – Ti-magnetite, Anl – analcime, Phl - phlogopite, Di – diopside, Cb – carbonate. Below the microphotographs two plots show Al_2_O_3_ - Mg# and CaO – Mg# plots for bulk rock compositions of central and marginal parts of the dykes, with arrows that lead from the margin to the core. The background for the major element diagrams are plots from Fig. 8 of [1], where red symbols denote Kandalaksha, green symbols for Kandaguba, blue symbols for Turiy Mys, small symbols and grey fields mark bulk compositional field for the Khibina dykes and massif [6], [7]. The lower two plots are trace element bulk rock compositions of central and marginal parts of the dyke normalized to the primitive mantle [8] and compared with the OIB patterns [8].

Tables 2 and 3 are output tables from Rhyolite-MELTS software used to model crystal fractionation of the dykes from feasible parental melts and various P, T, fO_2_, X CO_2_, X H_2_O parameters.

Table 2 provides compositions of evolved melt during equilibrium or fractional crystallization for samples Turiy Mys 19TR-19, TR-09; TR-30; Kandalaksha P408, 18KL-05, 19KL-21; Kandaguba 19KL-39 at various pressures and temperatures. The data are given as a single excel Raw data file with 5 separate spreadsheets (Electronic Supplementary Material 2) and also provided as five PDF tables online.

Table 3 entitled “Evolution of modal mineralogy and fluid composition during equilibrium or fractional crystallization for samples from Turiy Mys, Kandalaksha and Kandaguba” lists crystallizing phases modeled at various P-Ts, at the specified crystallization conditions. The data are given as a single excel Raw data file with 3 separate spreadsheets (Electronic Supplementary Material 3) and also provided as three PDF tables online.

## 2 Materials and Methods

We collected samples of Devonian dykes of lamprophyres, alkaline volcanic rocks and carbonatites in the Kola Alkaline Carbonatite Province. Thin sections of 85 dyke samples were examined optically and on a JEOL JSM-6480LV scanning electron microscope (Geology Department, the Lomonosov Moscow State University, Moscow, Russia). Minerals were quantitatively analyzed on the same SEM using the energy dispersive X-ray spectrometry at 20 kV, beam current 20 ηA, beam diameter 1–2 µm, 10 s peak counting time for major elements and 20–40 s for trace elements. Minerals in sample 19KL-19 were analyzed on a JEOL JXA-8200 equipped with five-wavelength spectrometers at the Institute of Geology of Ore Deposits, Petrography, Mineralogy and Geochemistry (IGEM RAS, Moscow) at the same analytical conditions. The maximum errors were ± 10 rel.% for 1–5 wt% elemental concentrations, ±5 rel.% for 5–10 wt% and ≤ 2% for >10 wt%. The minimum detection limits (MDLs) for elements ranged between 0.1 and 0.3 wt%. Both instruments were calibrated daily using both natural and synthetic standards, and measurements were corrected with the JEOL ZAF correction routine.

Bulk major element oxide compositions for 66 samples were determined by X-ray fluorescence (XRF) spectroscopy at IGEM RAS using a Philips Analytical B.V. PW-2400 spectrometer. The fused glass disks were prepared by melting 0.3 g of the powdered sample with 3 g of lithium tetraborate in an induction furnace. Analytical precision and accuracy were typically better than 1–5% for elements of concentrations greater than 0.5 wt% and <12 rel% for the remaining elements.

Trace element concentrations in 62 samples were determined by Laser Ablation Inductively Coupled Plasma Mass Spectrometry (ICP-MS) at the Institute of Microelectronics Technology and High Purity Materials (RAS, Chernogolovka) using analytical protocols of [3]. The rock powders were treated with inorganic acids in a sealed autoclave with the sample decomposition monitored by addition of ^161^Dy. MDLs were 0.02–0.03 ppm for REE, Hf, Ta, Th, and U; 0.03–0.05 ppm for Nb, Be, and Co; 0.1 ppm for Li, Ni, Ga, and Y; 0.2 ppm for Zr; 0.3 ppm for Rb, Sr, and Ba; and 1–2 ppm for Cu, Zn, V, and Cr. The spectrometer was calibrated against standards GSP-2, BM, SGD-1А, and ST-1. The 1σ analytical accuracy was better than 0.3 rel.% for elements with concentrations up to 5 x MDL and 0.15 rel.% for the remaining elements.

The Rhyolite-MELTS software package (version 1.2.0, [2]) was used to constrain conditions of crystallization and melt evolution of the studied dykes. The modeling was carried out at *T* = 1400–820 °C, *P* = 1.3 GPa- 10 MPa, for the most magnesian respective rock types, at Δlog fO_2_ (QFM)=0–2, overall fluid content 7 wt%, CO_2_/H_2_O ratios from 2/5 to 5/2, at equilibrium and fractional crystallization conditions.

**Fig. 8:**
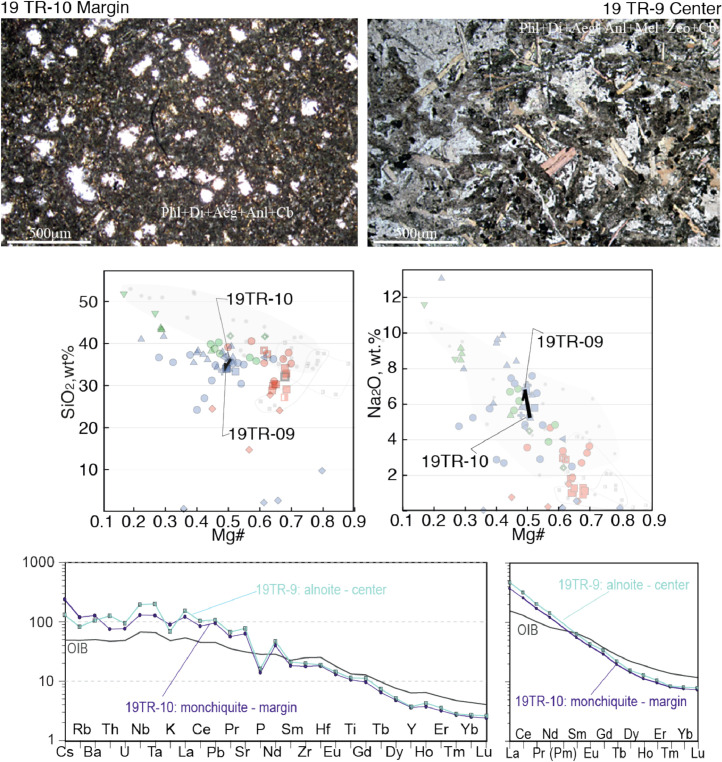
Contrasting petrography, major and trace element compositions of central (19TR-9) and marginal parts (19TR-10) of an alnoite monchiquite dyke. The upper microphotographs are optical plain polarized light microscopic images. Mineral abbreviations are Phl- phlogopite, Di diopside, Aeg aegirine, Anl analcime, Cb carbonate, Mel- melilite, Zeo-zeolite. Below the microphotographs two plots show SiO2 - Mg# and Na2O Mg# plots for bulk rock compositions of central and marginal parts of the dykes, with arrows that lead from the margin to the core. The background for the major element diagrams are plots from Fig. 8 of [1], where red symbols denote Kandalaksha, green symbols for Kandaguba, blue symbols for Turiy Mys, small symbols and grey fields mark bulk compositional field for the Khibina dykes and massif [6], [7]. The lower two plots are trace element bulk rock compositions of central and marginal parts of the dyke normalized to the primitive mantle [8] and compared with the OIB patterns [8]

## Ethics Statement

The authors declare that the work described is original and has not been submitted elsewhere for publication. No conflict of interest exists in this submission.

## CRediT Author Statement

**A.A. Nosova:** Conceptualization, Formal Analysis, Visualization, Supervision, Writing – original draft, Project administration; **M.G. Kopylova:** Conceptualization, Visualization, Supervision, Writing – original draft, Writing – reviewing & editing, Funding Acquisition; **L.V. Sazonova:** Investigation, Supervision; **A.A. Vozniak:** Investigation, Data curation; **N.M. Lebedeva:** Resources, Validation, Data curation; **G.D. Volkova:** Investigation, Visualization, Writing – original draft; **E.V Peresetskaya:** Investigation.

## Declaration of Competing Interest

The authors declare that they have no known competing financial interests or personal relationships which have or could be perceived to have influenced the work reported in this article.

## Appendix Supplementary materials

Image, application 1

Image, application 2

Image, application 3
